# Primary* Histoplasma capsulatum* Enterocolitis Mimicking Peptic and Inflammatory Bowel Disease

**DOI:** 10.1155/2016/7139573

**Published:** 2016-10-12

**Authors:** Rahman Nakshabendi, Andrew C. Berry, Daisy Torres-Miranda, Francis Daniel LaBarbera, Ozdemir Kanar, Ahmad Nakshabandi, Imad Nakshabendi

**Affiliations:** ^1^Division of Gastroenterology, University of Iowa, Iowa City, IA, USA; ^2^Department of Medicine, University of South Alabama, Mobile, AL, USA; ^3^Department of Medicine, University of Florida College of Medicine-Jacksonville, Jacksonville, FL, USA; ^4^Division of Gastroenterology, Florida Hospital Tampa, Tampa, FL, USA

## Abstract

In immunocompromised patients, histoplasmosis may present as disseminated disease. We present a 52-year-old Caucasian male with symptoms of dyspepsia, postprandial epigastric pain, nausea, and nonbloody diarrhea. Upper and lower gastrointestinal endoscopies were suspicious for inflammatory bowel disease (IBD); however, biopsies were consistent with histoplasmosis, specifically in the duodenum.

## 1. Introduction

*Histoplasma capsulatum* is a dimorphic fungus that infects by inhalation of spores and can disseminate to the gastrointestinal system. Immunocompetent patients that acquire histoplasmosis generally have a self-limited respiratory infection [1, 2]. Patients who are immunosuppressed are unable to develop effective cell-mediated immunity against the organism and are likely to manifest symptomatic disease during the period of acute dissemination. Gastrointestinal histoplasmosis (GIH) is common in patients with primary lung involvement, but it is very unusual to present with isolated involvement of the gastrointestinal system.

## 2. Case Presentation

A 52-year-old Caucasian male with no pertinent past medical history presented with dyspepsia, nausea, bloating sensation, and postprandial abdominal pain, associated with nonbloody watery-brown diarrhea for six weeks. He also reported decreased appetite and unintentional weight loss of about 30 pounds (13.6 kg) in the last two months. He denied use of NSAIDS, coffee, alcohol, tobacco, or illicit drugs, any recent travel, or sick contacts. Vital signs were within normal limits. Physical exam was unremarkable except palpation eliciting mild diffuse pain in all four quadrants without rebound tenderness and negative Murphy sign. The patient was started on proton pump inhibitors (PPI). After two weeks, his followup complete blood count, basic metabolic panel, and liver function panel were unremarkable. The patient reported no relief of symptoms with PPI and was referred for endoscopy, which the patient initially refused. He returned to clinic after 2 months with worsening symptoms and further loss of 10 pounds and agreed to have an endoscopy.

**Figure 1:**
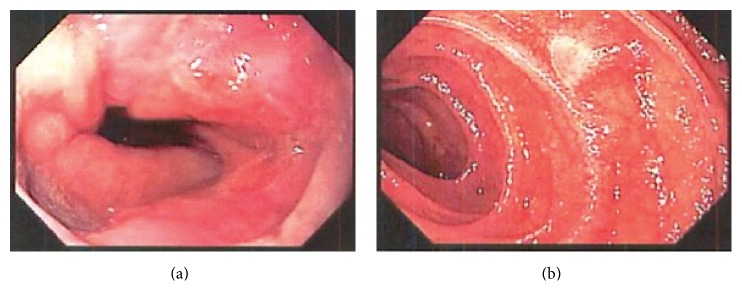
(a) Esophagogastroduodenoscopy (EGD) with squamocolumnar junction at 40 cm and evidence of esophagitis. (b) Esophagogastroduodenoscopy (EGD) showing scattered flat polypoidal lesions in the distal duodenum.

**Figure 2:**
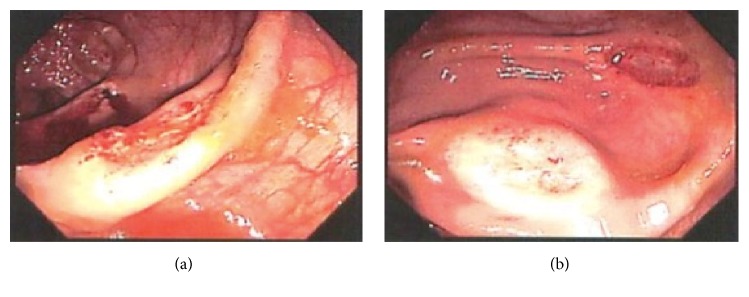
(a) Colonoscopy revealing several diffuse ulcerated focal lesions in the ascending colon and cecum. (b) Colonoscopy revealing several diffuse ulcerated focal lesions in the ascending colon and cecum.

EGD demonstrated the squamocolumnar junction at 40 cm with evidence of esophagitis (Figure 1(a)) and diffuse gastritis in the antrum extending to the body with a normal pylorus. A few scattered flat polypoidal lesions were found in the distal duodenum (Figure 1(b)). Biopsies were taken from the esophageal, gastric, and duodenal mucosa. Colonoscopy revealed several diffuse ulcerated focal lesions in the ascending colon and cecum (Figures 2(a) and 2(b)). Biopsies were obtained and sent for pathology. After endoscopic evaluation, suspicion was high for IBD, but no steroids were initiated. Distal duodenal biopsy was stained with Gomori's Methenamine Silver, revealing acute and chronic inflammation with macrophages and numerous yeast, consistent with* Histoplasma capsulatum*. Urine* histoplasma* antigen came back markedly elevated at >20 ng/mL, confirming the diagnosis. Our histoplasmosis urine antigen came back high-positive (not indeterminate) and protocol for high-positive is not set for reflux to confirmatory MiraVista (Indianapolis, Indiana) histoplasma quantitative enzyme immunoassay. Though unlikely, it is possible for other dimorphic fungal pathogens to infect macrophages and potentially cross-react with the urine antigen test (e.g.,* Blastomyces*,* Paracoccidioides*). However, our high-positive urine antigen test coupled with immunohistochemical evidence of histoplasmosis fully supports the diagnosis. No metaplasia or carcinoma was identified and no organisms were identified on an immunohistochemical stain for* Helicobacter*. At this stage, HIV screening was ordered and returned positive, with CD4 count of 76 cells/mm^3^. The patient was started on itraconazole therapy, as well as prophylactic trimethoprim/sulfamethoxazole (TMP/SMX), as his CD4 count was under 200. He will be seen in an outpatient clinic for initiation of ART.

## 3. Discussion

Patients with GIH are usually asymptomatic and diagnosis is often made incidentally or in autopsy finding [1]. It most commonly presents with fever, bloody diarrhea, abdominal pain, and hepatosplenomegaly on exam [3, 4]. Some case reports revealed patients presenting with odynophagia or dysphagia as a result of extrinsic compression from mediastinal adenitis or from mediastinal fibrosis as seen in the primary pulmonary manifestation of histoplasmosis. On endoscopic evaluation, involvement has been seen to occur from the mouth to the rectum. The upper gastrointestinal tract can have oropharyngeal ulceration, perforation, and obstruction [3]. Gastric involvement can range from patchy or continuous superficial ulcerations to deep ulcers with or without perforation [5]. Small bowel involvement is commonly characterized as segmental inflammation and ulceration. The majority (90%) of lesions involve the lower GI tract, most commonly the ileocecal region or colon [4, 6], likely due to this region's high presence of gut-associated lymphoid tissue (GALT), serving as potential entry sites for macrophages filled with* H. capsulatum* yeasts [4, 6]. In the largest case series of GIH in AIDS patients. Less than 4% of AIDS patients with GIH demonstrate duodenal disease, further confirming the unusual aspect of our patient's presentation [6].

In stable patients, such as the patient in our case, itraconazole treatment for 6 to 18 months may be the initial choice [1, 2]. Prophylaxis with itraconazole is recommended in patients with HIV and CD4 cell counts <150 cells/mm^3^ in specific areas where the incidence of histoplasmosis is >10 cases per patient-years. Antigen levels should be monitored during and for one year after therapy to ensure that there are no relapses. Our patient responded adequately to itraconazole treatment. It is imperative to wait for pathology results before initiating treatment for possible IBD with steroids, as it may exacerbate infectious processes in AIDS patients.

## 4. Conclusion

Disseminated histoplasmosis should be considered in immunocompromised patients, regardless of pulmonary symptoms, in endemic or nonendemic areas.* Histoplasma capsulatum* can mimic an innumerable amount of syndromes, from other infectious etiologies like tuberculosis to malignancy, PUD, and IBD, leading to inappropriate therapies and unnecessary surgical interventions.

## Abbreviations

IBD: Inflammatory bowel disease
GIH: Gastrointestinal histoplasmosis
PUD: Peptic ulcer disease
TMP/SMX: Trimethoprim/sulfamethoxazole
ART: Antiretroviral therapy.
